# Home Office: Working from a Private Place

**DOI:** 10.1007/s12599-023-00797-9

**Published:** 2023-04-12

**Authors:** Claudia Mueller, Alexander Maedche, Gerhard Schwabe, Mark Ackerman, Volker Wulf

**Affiliations:** 1 University of Siegen, Siegen, Germany; 2 Karlsruhe Institute of Technology (KIT), Karlsruhe, Germany; 3 University of Zuerich, Zuerich, Switzerland; 4 University of Michigan, Ann Arbor, MI USA

The labor world is currently undergoing a major transformation. New forms of digitalization are giving rise to new socio-technical working arrangements. The COVID-19 pandemic has acted as a burning glass, favoring new models of working from home suddenly and without notice.

Thus, more research on the “home office” phenomenon and resulting scientifically grounded knowledge is urgently required that helps practitioners to advance future arrangements of good (hybrid) working conditions. Even beyond COVID-19, many office employees will continue to work, at least partly, from home. Although the degree of virtualization will continue to increase, a combination of physical and virtual presence can be expected. Hybrid forms of working will become part of our future private and professional life.

Working from home goes hand in hand with great potential, but at the same time also with risks. Recent experiences with home schooling and home office have triggered intensive discussions on potential positive and negative outcomes of co-locating work with private and schooling activities. There is a need to design digital technologies appropriately to support individuals, groups, and organizations in ways that increase productivity and wellbeing towards better work and life. Thus, pursuing a socio-technical paradigm for understanding and designing for the home office is essential.

This makes it all the more important to now closely analyze the experiences and lessons learned during the COVID-19 phase and to set the research agenda and development of working practices from home for the future.

The six contributions of this special issue aim to look closely at the line drawn between organizational work practice and work from home practices. They help to better understand and open up the design space that requires careful maneuvering. With new concepts and dedicated investigations, the papers in this special issue contribute to meticulously pursuing, deepening and condensing the negotiation of this new field of design in order to make good working relationships and situations future-proof. They provide important insights and inspiration to accompany the “new normal of work” from the interdisciplinary perspective of business and information systems engineering. The contributions approach the important coordinates in the field from different points of view and help to open up the space that needs to be illuminated in more detail for the future.

Lena Waizenegger, Kai Schaedlich, and Bill Doolin inspect socio-material practices that delineate home office work and make visible how home office workers appropriate socio-material environments during temporal and longer-term breakdowns. Their findings identify important aspects that employers need to consider to ensure that socio-material factors are and remain conducive to home office practices.

Vanessa Kohn, Muriel Frank, and Roland Holten explore factors influencing the personal digital resilience of home office workers in connection with the forced transition to remote socio-technical structures during the lock-down phases. In their study of home office workers' personal sentiments, they show that positive experiences with telework are expressed when the adaptation of socio-technical systems in particular has been successfully implemented. Their analyses show that the expression of negative experiences is especially related to the lack of organizational structures and of the space for individual exchange of experiences.

Johannes Erhardt and Isabella Seeber focus their research on boundary setting practices of home office workers and explore the concepts of flexible and permeable boundaries and how they can be aligned well with digital workplace tools (DWT) to support job satisfaction. Particularly exciting are their findings on how DWT tools can positively moderate conflictual situations that appear in a variety of forms on the continuum between flexibility and permeability. They also point out the special responsibility of managers, who should ideally carry out the introduction and implementation in participatory processes with the home office workers, in order to establish clear lines of responsibility and accountability for the use of DWP tools.

As a result of their study, Julian Marx, Stefan Stieglitz, Felix Brünker, and Milad Mirbabaie propose a new archetype for the home office worker which has not yet received sufficient attention besides established models, such as the corporate employee and the digital nomad. With the concept of the "corporate nomad" they create a model to which they assign a number of new identity attributions, which are linked to new socio-spatial configurations that can better capture and describe the practices of the home office in the corporate context.

Christoph Weinert and Tim Weitzel analyze different dimensions of potential conflicts that may arise for home office workers regarding their personal life and work (LWC), e.g., in terms of time, stress, and behavior. They investigate how LWC influences job outcomes among teleworkers and how the IT environments influenced this relationship during the COVID-19 pandemic. Their study particularly highlights the role of the available IT infrastructure and how it relates to different types of telework.

The longitudinal study by Julia Lanzl is directed at exploring technostress factors in domestic work settings and how they are subject to factors which minimize or increase them. Technostress is associated with less job satisfaction, lower productivity, and a higher risk of burnout. The study analyzes in particular the relationship between social support dimensions as technostress inhibitors and technostress creators, presents possible lines of conflict and gives recommendations for organizational boundary management.

The special issue is complemented by an interview with Prof. Dr. Bettina Kohlrausch, Academic Director, Institute of Economic and Social Research (WSI) at Hans-Böckler-Foundation. The interview shows current tendencies in the development of home office scenarios and points out many blind spots that need to be further developed in the future, such as legal aspects in relation to occupational health and safety. Overall, the interview confirms general findings on the advantages and disadvantages of home office work and points out in particular that the potential of home office work is far from fully explored.

To summarize, it is clear that the COVID-19 pandemic has opened an organizational and socio-digital window and accelerated implementations that are now by no means less interesting or even fully mature. Working from home will keep many practitioners and researchers busy for a long time to come, and we hope that the contributions of this special issue will form solid foundations to ground and inspire further research.

We would like to express our profound gratitude to all the authors, our interviewee Prof. Kohlrausch, as well as the numerous associate editors and reviewers who have contributed their time and expertise so generously to the research presented in this special issue. A very special thank you goes to the Business and Information Systems Engineering team that made this special issue possible.

